# *Oenanthe Javanica* Extract Protects Mouse Skin from UVB Radiation via Attenuating Collagen Disruption and Inflammation

**DOI:** 10.3390/ijms20061435

**Published:** 2019-03-21

**Authors:** Young Her, Bich-Na Shin, Yun Lyul Lee, Joon Ha Park, Dae Won Kim, Ki Seob Kim, Hyunjung Kim, Minah Song, Jong-Dai Kim, Moo-Ho Won, Ji Hyeon Ahn

**Affiliations:** 1 Department of Dermatology, Kangwon National University Hospital, Kangwon National University School of Medicine, Chuncheon, Gangwon 24289, Korea; young6078@hanmail.net; 2 Department of Physiology, College of Medicine, Hallym University, Chuncheon, Gangwon 24252, Korea; tlsqlcsk21@nate.com (B.-N.S.); yylee@hallym.ac.kr (Y.L.L.); 3 Department of Biomedical Science and Research Institute for Bioscience and Biotechnology, Hallym University, Chuncheon, Gangwon 24252, Korea; jh-park@hallym.ac.kr; 4 Department of Biochemistry and Molecular Biology, and Research Institute of Oral Sciences, College of Dentistry, Gangnung-Wonju National University, Gangneung, Gangwon 25457, Korea; kimdw@gwnu.ac.kr; 5 Da Rum & Bio Inc., Chuncheon, Gangwon 24232, Korea; drnbio@naver.com; 6 Knotus Co. Ltd., Incheon 406-840, Korea; nicolehkim@naver.com; 7 Center for Virus Research and Testing, Korea Research Institute of Chemical Technology, Daejeon 34114, Korea; zlscydn@naver.com; 8 Division of Food Biotechnology, School of Biotechnology, Kangwon National University, Chuncheon, Gangwon 24341, Korea; jongdai@kangwon.ac.kr; 9 Department of Neurobiology, School of Medicine, Kangwon National University, Chuncheon, Gangwon 24341, Korea

**Keywords:** anti-inflammation, *Oenanthe javanica* extract, photoprotection, skin, ultraviolet B

## Abstract

In recent years, the use of botanical agents to prevent skin damage from solar ultraviolet (UV) irradiation has received considerable attention. *Oenanthe javanica* is known to exert anti-inflammatory and antioxidant activities. This study investigated photoprotective properties of an *Oenanthe javanica* extract (OJE) against UVB-induced skin damage in ICR mice. The extent of skin damage was evaluated in three groups: control mice with no UVB, UVB-exposed mice treated with vehicle (saline), and UVB-exposed mice treated with 1% extract. Photoprotective properties were assessed in the dorsal skin using hematoxylin and eosin staining, Masson trichrome staining, immunohistochemical staining, quantitative real-time polymerase chain reaction, and western blotting to analyze the epidermal thickness, collagen expression, and mRNA and protein levels of type I collagen, type III collagen, and interstitial collagenases, including matrix metalloproteinase (MMP)-1 and MMP-3. In addition, tumor necrosis factor (TNF)-α and cyclooxygenase (COX)-2 protein levels were also assessed. In the UVB-exposed mice treated with extract, UV-induced epidermal damage was significantly ameliorated. In this group, productions of collagen types I and III were increased, and expressions of MMP-1 and MMP-3 were decreased. In addition, TNF-α and COX-2 expressions were reduced. Based on these findings, we conclude that OJE displays photoprotective effects against UVB-induced collagen disruption and inflammation and suggest that *Oenanthe javanica* can be used as a natural product for the treatment of photodamaged skin.

## 1. Introduction

Skin aging is divided into intrinsic aging and photoaging caused by repeated exposure to ultraviolet (UV) radiation. Intrinsic aging is characterized by smooth, pale, and finely wrinkled skin, and photoaging includes coarse deep wrinkles, dyspigmentation, and telangiectasia [1]. Alterations in collagen fibers of the dermis are thought to cause the wrinkles observed in intrinsic aging and in photoaging due to an imbalance between collagen synthesis and degradation. Collagen is degraded by matrix metalloproteinases (MMPs). It is thought that UV-induced expressions of MMPs are related to the deep wrinkles seen in photoaged skin because UV light induces expressions of MMPs in the skin [2]. UV radiation induces expressions of MMP-1, MMP-3, and MMP-9 in the skin, and it has been suggested that MMP-1 initially disrupts collagen types I and III, after which MMP-3 and/or MMP-9 further degrade the disrupted collagen proteins [3].

Many agents that can suppress UV-induced expressions of MMPs and subsequent collagen degradation have been identified. Among these, various botanical agents prevent photoaging. For example, the treatment of reconstituted human skin (EpiDerm) with a pomegranate extract after UVB irradiation inhibits syntheses of MMPs (MMP-1, MMP-2, MMP-3, MMP-7, MMP-9, and MMP-12) [4], whereas a methanol extract of red alga Corallina pilulifera results in prominent reduction in expressions of UV radiation-induced MMP-2 and MMP-9 in a dose-dependent manner [5].

It has been reported that the exposure of the skin to UV radiation induces pathological changes, such as erythema, edema, scaling, changes in epidermal thickness, hyperpigmentation, tumorigenesis, and aging [6,7,8,9]. During UV radiation, the excessive production of reactive oxygen species (ROS) can disrupt the balance between pro-oxidant production and antioxidant defense [10]. ROS overproduction is an inducible factor caused by the expression of pro-inflammatory cytokines, such as tumor necrosis factor (TNF)-α and interleukin (IL)-1, during UVB irradiation. This triggers the expression of cyclooxygenase (COX)-2, leading to the formation of sunburned cells and inflammatory cell infiltration and activation [11,12,13]. In addition, TNF-α activates the expression of vascular cell adhesion molecule 1 in UVB-induced skin-damaged mice, leading to inflammation progression [14,15].

Water dropwort (*Oenanthe javanica*), a perennial herb with a characteristic aroma and taste, is widely distributed in East Asia and Europe. Water dropwort is consumed not only as a vegetable but also in medicinal preparations. Recent studies have shown that *Oenanthe javanica* has various pharmacological and biological activities including anti-inflammatory and antioxidant activities [16,17], and various supplements have been reported to attenuate skin damage after UV exposure by decreasing inflammation [18,19]. In this study, we determined whether an *Oenanthe javanica* extract (OJE) could inhibit expressions of MMPs and degradation of collagens as well as inflammatory cytokines in mice with UV-induced photodamage.

## 2. Results

### 2.1. OJE Suppresses UVB-Induced Skin Damage

**Figure 1:**
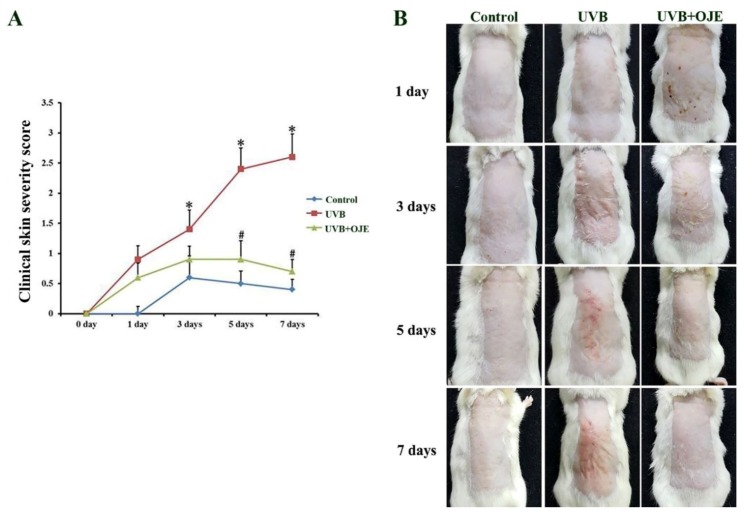
Effect of *Oenanthe javanica* extract (OJE) on UVB-irradiated skin lesion in mice. (**A**) The clinical skin severity score in the control, UVB, and UVB + OJE group at 1, 3, 5, and 7 days after UVB exposure. The UVB + OJE group shows significant attenuation in the severity score from 3 days after UVB irradiation. Bars indicate the standard errors of means; * indicates a significant difference from the control group (*P* < 0.05); # indicates a significant difference from the UVB group (*P* < 0.05; *n* = 10 per group). (**B**) Photos of dorsal skin samples in the control, UVB, and UVB + OJE group.

The effect of OJE on the UVB-exposed dorsal skin was assessed by analyzing levels of erythema/hemorrhage, scarring/dryness, edema, and excoriation/erosion (Figure 1). The clinical skin severity score was gradually and significantly increased in the UVB-exposed and vehicle-treated (UVB group) until 7 days after the exposure compared to the control group (Figure 1A,B). In contrast, the UVB-exposed and OJE-treated (UVB + OJE) group showed significant attenuation in the severity scores at 3 days after UVB irradiation and the ameliorative effect was sustained until 7 days after the exposure (Figure 1A,B).

### 2.2. OJE Prevents UVB-Induced Epidermal Thickening

**Figure 2:**
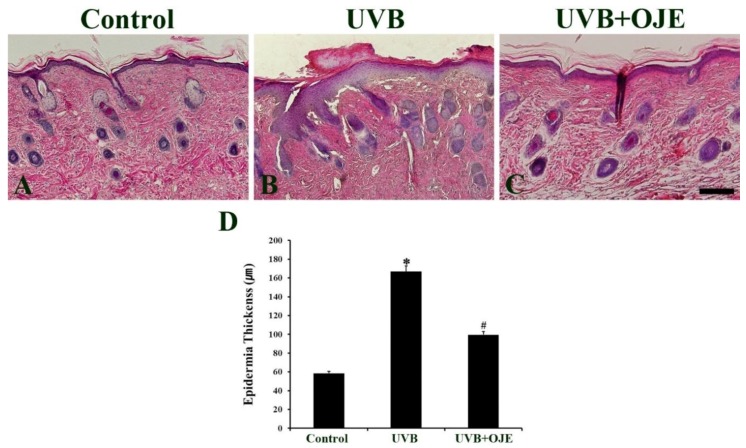
(**A**–**C**) Hematoxylin and eosin (H&E) staining of the dorsal skin in the control, UVB, and UVB + OJE group at 7 days after UVB exposure. UVB irradiation thickens the epidermis, while OJE prevents UVB-induced epidermal thickening. Scale bar = 100 μm. (**D**) The mean value of epidermal thickness in the control, UVB, and UVB + OJE group at 7 days after UVB exposure. * indicates a significant difference from the control group (*P* < 0.05); # indicates a significant difference from UVB group (*P* < 0.05; *n* = 10 per group). The data represent mean ± standard deviation.

The histological analysis of the dorsal skin was performed using hematoxylin and eosin (H&E) staining at 7 days after UVB exposure (Figure 2A–C). The epidermal thickness was calculated by performing measurement at 30 different sites from each section, and the mean value of the epidermal thickness for each group was calculated (Figure 2D). In the control group, the mean epidermal thickness was 50.88 μm (Figure 2A,D). In comparison, the UVB group showed a marked increase in epidermal thickness due to hyperplasia/hypertrophy (Figure 2B). In this group, epidermal thickness was increased by 3.41 fold compared to that in the control group (Figure 2D). However, UV-induced epidermal hypertrophy in the epidermis of the dorsal skin was significantly decreased in the UVB + OJE group compared to that in the UVB group (Figure 2C). In the UVB + OJE group, the epidermal thickness was 51.28% of the UVB group (Figure 2D).

### 2.3. OJE Protects Against UVB-Induced Dermal Collagen Fiber Loss

**Figure 3:**
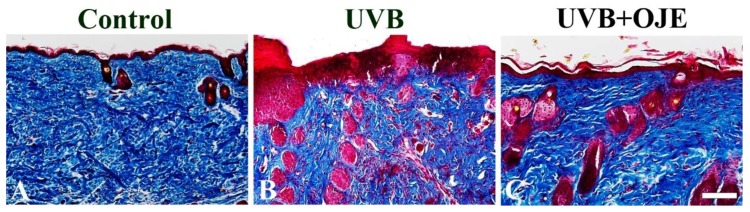
(**A**–**C**) Masson trichrome staining for the effect of OJE treatment on collagen fibers in the control, UVB, and UVB + OJE group at 7 days after of UVB exposure. In the UVB group, collagen fibers stained with blue dye are decreased in the dermis; however, in the UVB + OJE group, collagen fibers stained with blue dye are increased compared to the UVB group. Scale bar = 100 μm.

The effect of OJE treatment on the UVB-induced damage of dermal collagen fibers in the dorsal skin was evaluated using Masson trichrome staining at 7 days after UVB exposure (Figure 3). In the control group, collagen fibers stained with blue dye were abundantly found in the dermis of the dorsal skin (Figure 3A). However, in the UVB group, collagen fibers stained with blue dye were apparently decreased in the dermis due to the destruction of collagen fibers (Figure 3B). In contrast, in the UVB + OJE group, the reduction of collagen fibers was prominently ameliorated in the dermis compared to that in the UVB group (Figure 3C).

### 2.4. OJE Prevents UVB-Induced Decrease in Type I and III Collagen

**Figure 4:**
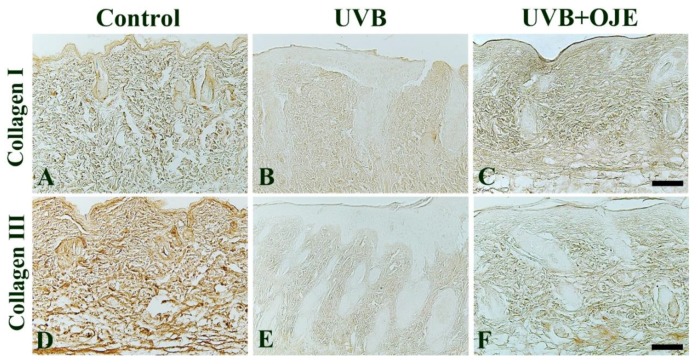
Immunohistochemistry for type I collagen (**A**–**C**) and type III collagen (**D**–**F**) in the dorsal skin of the control, UVB, and UVB + OJE group at 7 days after UVB exposure. In the control group, type I and III collagen immunoreactivity is strong throughout the dermis. In the UVB group, their immunoreactivity is markedly decreased, but immunoreactivity is higher in the UVB + OJE group than that in the UVB group. Scale bar = 100 μm.

The effect of OJE treatment on UVB-induced damage of collagen fibers in the dermis was evaluated using immunohistochemistry for type I and III collagen at 7 days after UVB exposure (Figure 4). In the control group, type I and III collagen immunoreactivity was strongly found in the dermis of the dorsal skin (Figure 4A,D). In the UVB group, type I and III collagen immunoreactivity were dramatically decreased throughout the dermis compared with that in the control group (Figure 4B,E). In addition, intracellular procollagen immunoreactivity in dermal fibroblasts in the control group displayed a highly dense regular arrangement, whereas the UVB group showed severe reduction and disruption in structural density (Figure 4B,E). However, in the UVB + OJE group, type I and III collagen immunoreactivity was apparently increased in the dermis compared to that in the control group (Figure 4C,F). 

**Figure 5:**
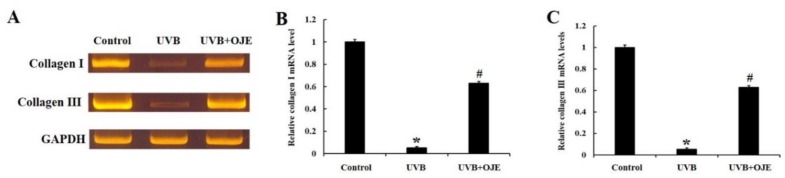
(**A**) Real-time polymerase chain reaction (RT-PCR) for mRNA of type I and III collagen in the dorsal skin of the control, UVB, and UVB + OJE group at 7 days after UVB exposure. (**B**,**C**) Relative levels of type I collagen (**B**) and type III collagen (**C**) mRNA in the dorsal skin. * indicates a significant difference from the control group (*P* < 0.05); # indicates a significant difference from the UVB group (*P* < 0.05; *n* = 10 per group). The data represent mean ± standard deviation.

The effect of OJE on mRNA expressions of type I and type III collagen was analyzed using quantitative real-time polymerase chain reaction (RT-PCR) analysis (Figure 5). Expressions of type I and type III collagen mRNA in the UVB group were significantly decreased in the dorsal skin compared to those in the control group (Figure 5A–C). However, expressions of type I and type III collagen mRNA in the UVB + OJE group were significantly increased compared to those in the UVB group (Figure 5A–C).

### 2.5. OJE Prevents UVB-Induced Induction of MMP-1 and MMP-3

**Figure 6:**
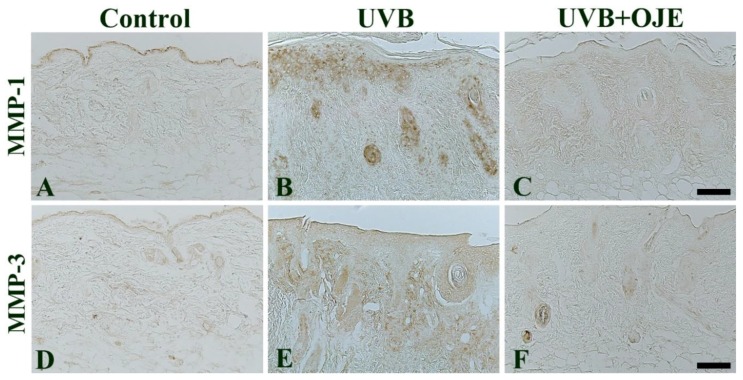
Immunohistochemistry for MMP-1 (**A**–**C**) and MMP-3 (**D**–**F**) in the dorsal skin of the control, UVB and UVB + OJE group at 7 days after UVB exposure. MMP-1 and MMP-3 immunoreactivity is increased in the UVB group, but their immunoreactivity is attenuated in the UVB + OJE group. Scale bar = 100 μm.

In the control group, MMP-1 and MMP-3 immunoreactivity was mainly found in the epidermis of the dorsal skin, and MMP-3 immunoreactivity was observed in the dermis (Figure 6A,D). MMP-1 and MMP-3 immunoreactivity in the dorsal skin of the UVB group was higher than that in the control group (Figure 6B,E). However, in the UVB + OJE group, MMP-1 and MMP-3 immunoreactivity was significantly decreased in the epidermis and dermis compared to that in the UVB group (Figure 6C,F).

**Figure 7:**
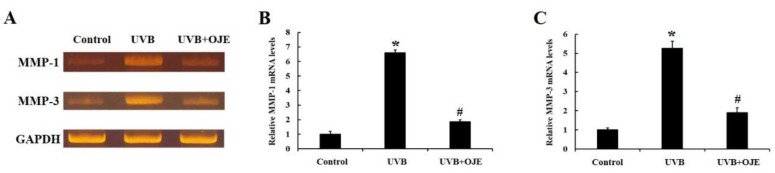
(**A**) Quantitative RT-PCR for effects of OJE on MMP-1 and MMP-3 mRNA expressions in the dorsal skin of the control, UVB, and UVB + OJE group at 7 days after UVB exposure. (**B**,**C**) Relative MMP-1 (**B**) and MMP-3 (**C**) mRNA levels in the dorsal skin. * indicates a significant difference from the control group (*P* < 0.05); # indicates a significant difference from the UVB group (*P* < 0.05; *n* = 10 per group). The data represent mean ± standard deviation.

The effects of OJE on MMP-1 and MMP-3 mRNA expressions were examined using quantitative RT-PCR analysis. The expressions of MMP-1 and MMP-3 mRNA in the UVB-exposed and 1% OJE-treated group were significantly decreased compared with those in the UVB-exposed skin-injured mice (Figure 7A–C). These results were consistent with the immunochemistry results.

### 2.6. OJE Prevents UVB-Induced Increase of TNF-α and COX-2 Protein Levels

**Figure 8:**
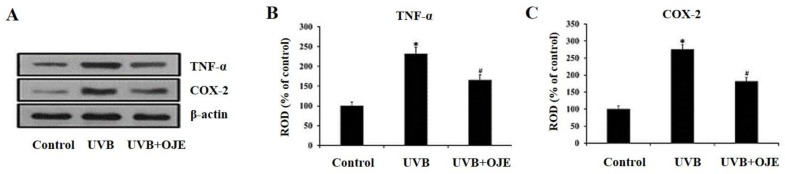
(**A**) Western blot analyses of TNF-α and COX-2 in the dorsal skin of the control, UVB, and UVB + OJE group at 7 days after UVB exposure. (**B**,**C**) Relative optical density (ROD) of TNF-α (**B**) and COX-2 (**C**) levels as percentage of immunoblot band in the dorsal skin. * indicates a significant difference from the control group (*P* < 0.05); # indicates a significant difference from the UVB group (*P* < 0.05; *n* = 10 per group). The data represent mean ± standard deviation.

Western blot analysis was performed to examine changes in TNF-α and COX-2 protein levels in the dorsal skin of the UVB group (Figure 8A). In the UVB + OJE group, the TNF-α protein level was significantly decreased compared to that in the UVB group (Figure 8B). Similarly, the COX-2 protein level in the UVB + OJE group was significantly lower than that in the UVB group (Figure 8C).

## 3. Discussion

Skin is the largest organ in the human body and acts as a primary physical barrier to the environment. Thus, skin is exposed to UV light through solar radiation. Three main subtypes of UV radiation are UVA (320–400 nm), UVB (280–320 nm), and UVC (200–280 nm) [20]. UVB radiation is the most active solar light component, and it is a thousand times stronger than UVA radiation. Thus, UVB radiation is known as “sunburn ray.” UVB radiation alters functions and structures of various proteins and genes, resulting in skin damage [21,22]. Scientific investigations have indicated that the beneficial effects of plants are due to the presence of chemical compounds known as phytochemicals including phenols, coumarins, lignans, essential oils, monoterpenes, glycosides, alkaloids, carotenoids, flavonoids, organic acids, and xanthines [23]. *Oenanthe javanica*, which had been used in our studies, has strong antioxidant properties via scavenging ROS in the liver and brain [16,17]. As described in the Introduction section, researchers have shown variable therapeutic effects of OJE; however, the photoprotective effect of OJE has not been elucidated, although such extract contains abundant phenolic compounds.

Previous studies have reported that major aspects of skin damage induced by UV light include epidermal thickening and changes in extracellular matrix proteins (ECM) in the dermis, such as collagen and elastin remodeling. In this study, we found that topical OJE application to UVB-damaged mouse skin displayed a positive effect on gross change in the dorsal skin. Namely, OJE alleviated change in epidermal thickness caused by UVB exposure. Moreover, in the mouse dermis, topical OJE application prevented an increase in mRNA expressions of MMP-1 and MMP-3 and a decrease in mRNA expressions of collagen I and III after UV exposure. In addition, we found that OJE treatment ameliorated UV-induced epidermal damage, decreased UV-induced MMP-1 and MMP-3 expressions, and increased collagen I and collagen III productions. Our results are in line with a previous study that showed that expressions of ECM proteolytic enzymes (MMP-1, MMP-2, MMP-3, and MMP-9) increased after UV radiation in normal human epidermis [4]. In addition, Röck et al. (2011) have reported that UV exposure impairs collagen synthesis, primarily through the downregulation of type 1 procollagen expression, and it affects type III procollagen [24]. In this regard, there is a possibility that OJE may improve activities of transcription factors related to procollagen expression, although the mechanisms underlying the enhancement of collagen production and inhibition of MMPs require clarification. Anyway, these findings show a potential value of OJE as a natural product for the treatment of photodamaged skin.

Inflammation, erythema, edema, sunburn, hyperplasia, and immunosuppression in the skin results from solar UVB radiation, which together have been implicated in the development of skin cancer [25]. Acute UVB exposure-mediated adverse reactions are characterized by activation of a number of cell signaling molecules [26]. It has been reported that UVB radiation activates proinflammatory markers such as TNF-α, IL-6, inducible nitric oxide synthase (iNOS), and COX-2, and it initiates an intracellular signaling cascade [27]. In addition, Adhami et al. (2003) have reported that proinflammatory and inflammatory molecules, such as TNF-α and COX-2, promote the release of NF-κB, and they induce various pathophysiologic changes [27]. NF-κB propagates inflammation-induced signals and aggravates skin damage via increasing ROS production [28]. In agreement with the previous results, in our current study, we found that UVB exposure significantly induced COX-1 (an inflammatory mediator) and TNF- α (a proinflammatory cytokine) expressions. However, OJE significantly downregulated levels of COX-1 and TNF-α in the mouse dorsal skin after UVB exposure.

Previously, natural products have been demonstrated to attenuate skin damage after UV exposure due to their health benefits, namely, their secondary metabolites such as phenols, coumarins, and glycosides. Among these metabolites, phenolic compounds have extensively been investigated for their strong antioxidant and anti-inflammatory activities [29,30]. Although they make up a small portion of compounds in plants, phenolic compounds exhibit a wide variety of beneficial biological activities in mammals [23,31]. Topical applications of phenolic compounds with high antioxidant and anti-inflammatory activities can ameliorate UV-induced photoaging. It has been reported that *Oenanthe javanica* contains caffeic acid and chlorogenic acid as major phenolic compounds [32]. These two phenolic acids have been reported to inhibit UVB-induced inflammation and to display antioxidant properties [33,34]. In addition, caffeic acid treatment blocks UVB-induced collagen degradation in human skin fibroblasts [32]. Chlorogenic acid, an ester of caffeic acid with quinic acid, is one of the most abundant polyphenols in human diets. It attenuates UVB-mediated oxidative stress in human keratinocytes and inhibits UVB-induced inflammation [35]. It has been reported that excessive inflammatory mediators and proinflammatory cytokines induce collagen degradation by enhancing expressions of MMPs and preventing the expression of procollagen [36]. Taken together, it is likely that special compounds of OJE may be closely related to ameliorating collagen disruption and inflammation in the mouse dorsal back skin after UVB exposure.

In conclusion, our current study implies that the photoprotective effect of OJE may be associated with inhibition of UVB-induced collagen disruption and inflammation, although additional studies are required to uncover exact pathways underlying its anti-inflammatory effect.

## 4. Materials and Methods

### 4.1. Experimental Animals and UVB Irradiation

All animal care was performed in accordance with the Guide for the Care and Use of Laboratory Animals and was approved by the Animal Care and Use Committee of Kangwon National University of Korea. Because the UVB-induced skin damages were not significantly affected by gender, a total of 30 male ICR mice weighing 32‒38 g at 8‒9 weeks of age were purchased from the Experimental Animal Center, Hallym University, Chuncheon, Republic of Korea. The animals were housed under conventional temperature (23 °C), humidity (60%), and 12-h light/dark cycle conditions and given free access to food and water. All experimental procedures for the handling and care of animals were conducted in compliance with the current international laws and policies (NIH Guide for the Care and Use of Laboratory Animals, NIH Publication No. 85-23, 1985, revised 1996). All experiments were performed to minimize the number of animals used and their suffering due to the procedures used in the present study. The source of UVB was a UVM-225D Mineralight UV Display Lamp (UVP, Phoenix, AZ, USA), and the intensity of radiation to which ICR mice were exposed was 150 mJ/cm^2^.

### 4.2. Preparation and Treatment of OJE 

*Oenanthe javanica* specimens were collected during 1 week in Kangwon Province, Republic of Korea, and extracted with 10 vol. (*v*/*w*) of 70% ethanol at 70 °C for 4 h. After repeating the extraction three times and removing the acetone under a vacuum, the extract was filtered through Whatman filter paper (No. 2), concentrated using a vacuum evaporator, and eluted with a freeze-drier. The extraction yield was 15.5%.

For the UVB protection experiment, mice were divided into three groups (*n* = 10 per group). The experimental groups were as follows: (1) control mice, (2) UVB-exposed and vehicle-treated mice, and (3) UVB-exposed and 1% OJE-treated mice. The dorsal hairs of the mice were shaved 3 days before UVB exposure. 

UVB irradiation exposure was applied at 150 mJ/cm^2^ for 10 min for 1 day using the UVM-225D Mineralight UV display lamp. OJE was dissolved in a solution of propylene:ethanol:distilled water (DW) = 5:3:2, and 1% OJE-treated mice received 200 μL of topical applications two times daily onto the dorsal skin for 7 days following UVB exposure. The control and UVB-exposed and vehicle-treated mice received topical applications of solvent which dissolved OJE (a solution of propylene:ethanol:DW = 5:3:2).

### 4.3. Observation of Clinical Severity of Skin Injury 

The clinical severity of mouse skin lesion (dermatitis) was scored using the macroscopic diagnostic criteria normally used when assessing the severity of dermatitis in humans [37]. In this study, the severity of dermatitis was evaluated four times a week by two independent dermatologists. The development of various skin conditions including erythema/hemorrhage, scarring/dryness, edema, and excoriation/erosion was scored as 0 (none), 1 (mild), 2 (moderate), and 3 (severe). The sum of the individual score was taken as the dermatitis score.

### 4.4. Tissue Processing for Histology 

For histological analysis, the mice (*n* = 10 each group) were anesthetized with sodium pentobarbital (30 mg/kg, intraperitoneal injection; JW Pharm. Co., Ltd., Seoul, Korea) at 7 days after UVB exposure and perfused transcardially with 0.1 M phosphate-buffered saline (PBS; pH 7.4), followed by 4% paraformaldehyde in 0.1 M phosphate buffer (PB; pH 7.4). Their dorsal skin tissues were fixed in paraformaldehyde, embedded in paraffin, sectioned into 7-µm slices (Leica, Wetzlar, Germany), and stained for histological analysis according to our published procedure [38].

### 4.5. H&E Staining 

Pathological changes of the dorsal skin in each group were assessed using H&E staining. Briefly, the sections were stained with H&E, dehydrated by immersion in serial ethanol baths, and then mounted with Canada balsam (Kanto Chemical, Tokyo, Japan) [17].

### 4.6. Masson Trichrome Staining

To examine changes of collagens in the dorsal skin, the sections were performed using Masson trichrome staining as described by Ahmet et al. [39]. In brief, the sections were placed in Biebrich scarlet-acid fuchsin solution (Sigma-Aldrich, St. Louis, MO, USA; Merck Millipore, Darmstadt, Germany) and aniline blue (Sigma-Aldrich; Merck Millipore). The stained images were acquired using a light microscope (BX53; Olympus, Hamburg, Germany) equipped with a digital camera (DP72; Olympus) connected to a PC monitor. Whole images of the dorsal skin were merged using image analyzing system Optimas version 6.5 (CyberMetrics, Phoenix, AZ, USA).

### 4.7. Immunohistochemistry for Type I and III Collagen and Interstitial MMP-1 and MMP-3

Immunohistochemistry was carried out using antibodies against type I collagen, type III collagen, MMP-1, and MMP-3 to investigate changes of collagen and interstitial collagenase expressions in the sections. The sections were incubated with 0.3% hydrogen peroxide in PBS for 30 min, followed by 10% normal donkey or goat serum (Vector Laboratories, Inc., Burlingame, CA, USA) in 0.05 M PBS for 30 min. The sections were incubated with rabbit anti-type I collagen (diluted 1:400; Abcam, Cambridge, UK), mouse anti-type III collagen (diluted 1:300; Abcam), rabbit anti-MMP-1 (diluted 1:300; Abcam), and mouse anti-MMP-3 (diluted 1:300; Abcam). Thereafter, the tissues were exposed to biotinylated donkey anti-rabbit IgG, goat anti-mouse IgG, and streptavidin peroxidase complex (Vector Laboratories) and visualized with 3,3′-diaminobenzidine tetrachloride (Sigma-Aldrich, Darmstadt, Germany). After dehydration, the sections were mounted with Canada balsam (Kanto, Tokyo, Japan). 

We performed quantitative analyses of the immunoreactivities against type I collagen, type III collagen, MMP-1, and MMP-3 using an AxioM1 light microscope (200× magnification; Carl Zeiss, Oberkochen, Germany) equipped with a digital camera (Axiocam) and connected to a PC monitor.

### 4.8. Quantitative RT-PCR Analysis

The expressions of type I collagen, type III collagen, MMP-1, and MMP-3 genes were examined using RT-PCR with primers corresponding to data in GenBank. The specific primers were chemically synthesized using a DNA synthesizer. Total RNA was extracted from the dorsal skin tissues using TRIzol^®^ reagent (Invitrogen, Carlsbad, CA, USA). The cDNA was synthesized from equal amounts of total isolated RNA with reverse transcriptase, and PCR was performed using a Quanti Tect SYBR Green PCR Kit (Qiagen, Germany). The cDNA was amplified using 60 cycles of denaturation (95 °C for 30 s), annealing (58 °C for 30 s), and extension (72 °C for 45 s) with primers. 

### 4.9. Western Blotting

Protein levels of TNF-α and COX-2 in the dorsal skin (*n* = 10 at each point in time) were analyzed according to our published procedure [17]. After sacrificing the animals, the tissues were homogenized in 50 mM PBS (pH 7.4) containing 0.1 mM ethylene glycol-bis (2-aminoethyl ether)-N,N,N′,N′ tetraacetic acid (pH 8.0), 0.2% nonidet P-40, 10 mM ethylenediaminetetraacetic acid (pH 8.0), 15 mM sodium pyrophosphate, 100 mM β-glycerophosphate, 50 mM NaF, 150 mM NaCl, 2 mM sodium or thovanadate, 1 mM phenylmethylsulfonyl fluoride, and 1 mM dithiothreitol (DTT). The skin tissues were separated using 4‒20% sodium dodecyl sulfate-polyacrylamide gel electrophoresis (SDS-PAGE) for 3 h, and the resolved proteins were transferred to a nitrocellulose membrane for 2 h at 40 V. Each membrane was incubated separately with the primary antibodies: rabbit anti-TNF-α (1:1000, Abcam) and rabbit anti-COX-2 (1:1000, Abcam) overnight at 4 °C. The membranes were washed with a washing buffer (137 mM NaCl, 2.7 mM KCl, 10 mM Na2HPO4, 2 mM KH2PO4, and 0.05% Tween-20) and exposed to peroxidase-conjugated goat anti-rabbit IgG (Santa Cruz Biotechnology, Santa Cruz, CA, USA) at a 1:1000 dilution at room temperature for 2 h. The membrane blots were developed using an enhanced luminol-based chemiluminescent (ECL) kit (Pierce Chemical, TX, USA).

### 4.10. Statistical Analysis

All data are presented as mean ± standard errors of mean. A multiple sample comparison was applied to test the differences between groups (one-way or multiple-way analysis of variance and Tukey’s multiple range test as a post hoc test using the criterion of the least significant differences). The analysis of variance was followed by a Bonferroni’s post-hoc test, using GraphPad Prism 5.01 software (GraphPad Software, Inc., La Jolla, CA, USA). A *P*-value < 0.05 was considered to indicate significance of the results.
